# Barriers and facilitators to targeted anxiety prevention programmes in families at risk: a qualitative interview study

**DOI:** 10.1007/s00787-020-01703-4

**Published:** 2020-12-21

**Authors:** P. J. Lawrence, K. Harvey, C. Williams, C. Creswell

**Affiliations:** 1 School of Psychology, University of Southampton, Southampton, UK; 2 School of Psychology and Clinical Language Sciences, University of Reading, Reading, UK; 3 Department of Experimental Psychology , University of Oxford, Oxford, UK; 4 Department of Psychiatry, University of Oxford, Oxford, UK

**Keywords:** Anxiety, Behavioural inhibition, Prevention, Treatment access, Risk

## Abstract

Anxiety disorders are the most common psychiatric disorder in children and young people. They can be prevented in those at risk, but families do not always take up opportunities to participate in prevention programmes. This qualitative study aimed to understand what families with children who were at prospective risk of anxiety disorders perceived to be the barriers to access to targeted anxiety prevention programmes, and to explore what would help facilitate access. We used Information Power to determine our sample size, and individually interviewed seven young people (14–17 years) who had anxiety disorders and their mothers, each of whom had pre-natal anxiety disorders. We transcribed all interviews and thematically analyzed them to identify perceived barriers and facilitators to targeted anxiety prevention programmes. Perceived potential barriers to access included possible negative consequences of anxiety prevention, difficulties in identifying anxiety as a problem and concerns about how professions would respond to raising concerns about anxiety. Possible facilitators included promoting awareness of anxiety prevention programmes and involvement of schools in promotion and delivery of prevention. Our findings illustrate that implementation of targeted anxiety prevention could be improved through (i) the provision of tools for parents to recognize anxiety in their children as a problem, (ii) promotion of awareness, as well as delivery, of anxiety prevention via schools and (iii) the involvement of parents and possibly adolescents in the intervention programme, but not younger children.

**Supplementary Information:**

The online version contains supplementary material available at 10.1007/s00787-020-01703-4.

## Introduction

Anxiety disorders are the most common psychiatric disorder in children and young people (CYP) [1]. They have an early age of onset—approximately 50% emerge by age 11 years [2], are often chronic [3], and are associated with a significant negative impact in multiple domains of life, including later psychiatric illness (depression, psychosis, substance abuse), school drop-out, educational under-attainment, victimization by peers and impaired life satisfaction [3–6]. Prevention could help minimize these negative impacts.

Anxiety disorders are preventable [7, 8]. Psychiatric prevention programmes are typically classified as ‘universal’, ‘selective’ or ‘indicated’ [9]. Universal prevention is provided to whole populations; selective prevention to those who show risk factors for a psychiatric disorder; and indicated prevention to those who show symptoms of the disorder. In a recent meta-analysis, Stockings et al. [10] found that universal prevention programmes effectively prevented anxiety disorders when measured at the end of treatment [relative risk (RR) = 0.25, 95% CI = 0.10–0.65], but not at follow ups between 6 and 18 months.

Crucially, it is unclear whether universal programmes have a clinically meaningful impact on those who most need them; i.e. those who are actually at risk of developing anxiety disorders [11]. For example, Stallard et al. [12] reported a randomized controlled trial (RCT) of universal anxiety prevention in schools in England for 9–10-year olds, where the prevention programme effectively reduced the anxiety symptom severity as compared to standard school provision when delivered by health (but not school) staff. Crucially, the pattern of the results suggested that, compared to usual school provision, children who did not have severe anxiety symptoms at baseline benefited from prevention, but the children who had the most severe anxiety symptoms at baseline did not benefit. These findings highlight that universal anxiety prevention might be ineffective for those who are most at risk of developing anxiety disorders.

However, there is evidence that targeted prevention programmes, that is, for CYP with particular risk factors (i.e. selective prevention) or already showing anxiety symptoms (i.e. indicated prevention) can be effective [13]. Hugh-Jones et al. [14] reported a meta-analysis of school-based indicated anxiety prevention programmes, where, when compared with controls, prevention effectively reduced anxiety symptoms at end of programme (*g* = − 0.28, 95% CI = − 0.050 to − 0.05) and at 6-month follow-up (*g* = − 0.35, 95% CI = − 0.58 to − 0.13).

Meta-analyses show that one such risk factor is parent anxiety disorders [15, 16]. In their meta-analysis of targeted anxiety prevention, Lawrence et al. [17] found that prevention programmes targeting children of parents with anxiety disorders effectively reduced the risk of onset of anxiety disorders in children (at the end of treatment, RR = 0.09, 95% CI = 0.02–0.16; and 1 year later, RR = 0.31, 95% CI = 0.17–0.45). Another risk factor for CYP anxiety disorders is the infant temperamental pattern of fear and withdrawal in unfamiliar situations, that is, behavioural inhibition (BI) [17]. Meta-analyses of longitudinal studies have shown, compared to children without BI, there is significantly increased risk of anxiety disorders in those with BI [18, 19]. To date, RCTs targeting CYP in light of BI have mixed selective prevention and early intervention (i.e. for children with BI—some of whom already have anxiety disorders at baseline, while others do not). Rapee et al., for example, targeted 3- to 5-year-old children, identified on the basis of BI, of whom 90% had an anxiety disorder at baseline [20, 21]. Rapee [22] found that, 11 years after the programme (at approximately 15 years of age) females who received the programme showed lower rates of internalizing disorders than those who were simply monitored. Further, in an RCT where 3–4-year olds were identified in the context of both parental anxiety disorders and BI, Kennedy et al. [23] found that, compared to a wait list control group, at 6-month follow-up, those in the prevention group had fewer anxiety disorders.

Different opinions have been expressed about implementing targeted anxiety prevention. For example, the most recent World Health Organization [24] report on prevention of mental disorders prescribed that “successful (prevention) programmes and policies should be made widely available”. However, in a cautionary note, Fox et al. [25] highlighted that while effective targeted prevention of anxiety disorders for inhibited preschoolers has been demonstrated [21–23], the majority of children with BI will not develop an anxiety disorder and so, targeted anxiety prevention programmes could inadvertently and incorrectly label children as anxious, fuel parental worry about their child and potentially create an opportunity cost by allocating resources this way.

Despite the promise of targeted anxiety prevention programmes, we know little about how to implement them [26, 27]. Key difficulties identified include low rates of attendance at, and engagement with, programmes, as well as parent reluctance to attend group-based programmes [26, 27]. Implementation of a combined anxiety prevention and early intervention programme also indicates that participants experience difficulties including managing the time commitment, although flexible access to an online programme appears to be popular [28]. Importantly, these findings come only from RCTs, where the samples comprise people who have obtained access to clinical programmes. Although this is informative, to support the development and implementation of targeted anxiety prevention, we need to know more about barriers and facilitators in at risk community samples who have not necessarily gained access to prevention programmes. To our knowledge, studies with such samples have not been reported in the literature.

Qualitative studies have examined young people’s and parents’ experiences of barriers and facilitators to access to treatment for common mental health problems outside RCTs. For example, Radez et al. [29] examined barriers and facilitators to treatment in a systematic review, and found four themes: individual CYP factors, such as limited knowledge of mental health problems including anxiety, social factors, such as embarrassment and perceived stigma; CYP perceptions of therapeutic relationships, such as issues of confidentiality and structural factors, such as financial cost. Qualitative studies of barriers and facilitators to access to treatment for anxiety disorders in particular [30, 31], have identified difficulties including identifying anxiety as a problem, anticipating that professionals would dismiss concerns and limited service provision; while factors facilitating access to treatment included raising awareness of how to seek help and trust in teachers at CYPs schools. Although many of these themes may also apply to prevention, this has not been examined to date. In line with the UK Medical Research Council guidance on developing complex interventions, qualitative approaches are important to understand barriers and facilitators to access as a key step in intervention development [32].

The current study aimed to explore what adolescents with anxiety disorders who, as infants, had been identified as at risk for anxiety disorders on the basis of maternal anxiety disorders and/or BI and their mothers (i) perceived to be the potential barriers to access to targeted anxiety prevention programmes and, (ii) what characteristics of targeted anxiety prevention programmes could help facilitate access to these programmes at any time between identification of risk and onset of disorder.

## Methods

### Ethical considerations

The University of Reading Research Ethics Committee approved the study (UREC 99/14; UREC 16/18). All participants Query received information sheets and provided written informed consent to participate in the study or, where under 16 years of age, written informed assent.

### Sample

Inclusion criteria for the present study sought to obtain a sample of CYP who had been assessed as infants to be at risk of anxiety disorders and gone on to develop an anxiety disorder. Thus, we required that (i) during infancy, mothers had been diagnosed with an anxiety disorder according to DSM-IV [33] or infants had been assessed as behaviourally inhibited or both (i.e. identifying infants at risk) and (ii) that adolescents had a current or past anxiety disorder according to DSM-5 [34]. We used criterion sampling [35] to purposively recruit from a sample of participants in a longitudinal cohort study [36].

#### Inclusion criterion (i)

In the first wave of the longitudinal study (1999–2004), 4000 women were screened for Social Anxiety Disorder (SAD) and Generalized Anxiety Disorder (GAD) at their routine 20-week antenatal screening appointments (using the Social Interaction and Anxiety Scale (SIAS), the Social Phobia Scale (SPS) [37] for SAD and the Penn State Worry Questionnaire (PSWQ) [38] for GAD). Trained postgraduate researchers conducted diagnostic psychiatric interviews with 427 mothers (304 whose screening scores suggested probable diagnoses) and discussed audio recordings of their interviews with a team of senior clinical researchers to confirm diagnoses. 152 mothers with anxiety disorders and 94 mothers without SAD or GAD were recruited to the longitudinal study. Socio-economic status was assessed using the UK National Statistics Socio-economic classification (NS-SEC) [39].

Behavioural inhibition (BI) was assessed in university laboratories when infants were 14 months of age, using seven frames of observation following Kagan’s procedures [17]. The first three frames were each one minute’s exposure to a mechanical dinosaur, the fourth frame was three minutes with novel toys in an unfamiliar room and the final three frames were each one minute with an unfamiliar adult female (first an approach phase, then a pick-up phase and finally a play phase). Children’s latencies to approach novelties and fearful or distressed behaviours in response to novelties were assessed in each frame, and were coded as BI or not in each frame, which yielded a total score on the scale of 0 to 7. Children with a score above three were classified as BI [40]. Two trained postgraduate researchers, blind to maternal group, scored videos. From the original sample of 246 dyads, twenty videos were independently second-scored by a third trained postgraduate researcher, and kappa was 1.0 (see [36] for details on recruitment to wave one and Table 1.)

**Table 1:** Demographics of baseline and current samples

	*N* (%)	
	Baseline at risk sample (*N* = 152)	Current sample (*N* = 7)
Low SES	19 (12.5)	2 (28.6)
Male	64 (42.1)	1 (14.3)
Ethnicity—White British	144 (94.7)	7 (100)

#### Inclusion criterion (ii)

In the current (third) wave of the longitudinal study (2016–2018), we sent invitations to participate in the study to all mother–adolescent dyads from the baseline sample who, in 2005–2009, had agreed to be contacted (*n* = 188). Of these, 34 mother–adolescent dyads consented to participate in follow-up assessments, 1 declined and we were unable to make contact with the remaining 211. Adolescents were aged between 14 and 17 years. Of the 34 dyads, seven met the inclusion criteria for the current study, and all were invited, by telephone, to participate; all agreed. While theoretical saturation is a commonly reported method to determine sample size in qualitative research [41], in this study, we adopted the concept of Information Power to inform our sample size [42]. This was because three characteristics of our study provide us with high information power: our aim was narrow rather than broad; our sample specificity was dense rather than sparse; and our dialogue was strong rather than weak.

The sample in the current study was entirely White British. All adolescents had been at risk in light of maternal anxiety disorders, and two also in light of BI. All had a diagnosis of at least one anxiety disorder. Four dyads reported a history of seeking professional help for the adolescent’s anxiety disorder, but none had been offered, nor sought access to, an anxiety prevention programme (participant characteristics are described in Table 2.)

**Table 2:** Participant characteristics

ID	Family SES	Mother	Adolescent						
		Age (years)	Age (years)	Gender	Risk factors		Current anxiety disorder(s)	Received treatment	Ethnicity
					BI	Maternal Anxiety			
1	Lower/supervisory and technical	47	17	F	Yes	Yes	SAD	Yes	White British
2	Intermediate occupations	50	17	M	Yes	Yes	SAD	No	White British
3	Higher/professional	54	16	F	No	Yes	SAD	No	White British
4	Lower managerial and professional	39	16	F	No	Yes	SAD	Yes	White British
5	Intermediate occupations	44	15	F	No	Yes	GAD, PD, SAD	No	White British
6	Higher/professional	49	15	F	No	Yes	SAD	Yes	White British
7	Lower managerial and professional	43	14	F	No	Yes	SAD	Yes	White British

NB All adolescent diagnoses were made at the same age as they undertook the interview in the current study

*BI *behavioural Inhibition, *GAD *Generalized Anxiety Disorder, *ID *identifier, *Maternal Anxiety *maternal anxiety disorder, *PD *Panic Disorder, *SAD *Social Anxiety Disorder, *SES *socio-economic status

### Procedure

For the current study, we conducted individual one-to-one semi-structured interviews with each of the seven adolescents and seven mothers. All interviews were conducted at a location of the participant’s choice (typically at home, but three at university), all but one interview was conducted face-to-face (with a mother via Skype at that participant’s request), and no one else was present at any interview. Interviews with adolescents ranged from 14 to 35 min (mean = 24 min) and with mothers from 27 to 46 min (mean = 39 min). PJL, an experienced male clinical psychologist and CW, a female UG psychology student conducted the interviews. Both were trained in qualitative methods. The study contributed to PJL’s doctoral research and CW undertook the interviewing as part of their UG research project. PJL had conducted the diagnostic assessments with adolescents and their mothers (see ‘Participant flow diagram’, supplementary materials) ahead of the current study. CW had no contact with participants prior to the current study. Before beginning each interview, interviewers explained that the aim of the study was to contribute to the development of accessible anxiety prevention programmes, as well as their reasons for doing the research. We developed a Topic Guide in collaboration with a Patient and Public Involvement (PPI) group using the results of studies examining barriers to access to prevention and treatment of anxiety disorders [e.g. 25,26,28] and our clinical experience. The Topic Guide sought to explore (i) what participants thought about the idea of preventing anxiety disorders (with the aim of understanding their views on targeted prevention of anxiety disorders and (ii) what they viewed to be barriers or facilitators to gain access to such programmes. We used the Topic Guide flexibly, to ensure that participants could describe issues that were incongruent with the prepared areas of questions [43]. Interviews lasted between 14 and 46 min. We made audio recordings and transcribed them verbatim (removing identifying information). We made Fieldnotes during each stage of the data collection and analysis. We did not return transcripts to participants for comment and did not invite participants to give feedback on our finalized themes, although they were offered/provided with a summary of our findings.

### Measures

#### Adolescent diagnostic interview

As part of the third wave of the longitudinal study, all adolescents and their mothers separately completed the Adolescent Diagnostic Interview Schedule-IV-Child and Parent Interviews (ADIS-C/P) [44]. These are semi-structured diagnostic assessment tools, specifically for anxiety disorders, widely used in the literature. We made amendments to the ADIS-C/P so that we could make diagnoses consistent with the criteria of the Diagnostic and Statistical Manual (DSM) DSM-5 [34], rather than DSM-IV [34]. As is standard, diagnoses were assigned based on either adolescent or mother report (that is, if either or both reported symptoms and interference meeting diagnostic criteria). All ADIS-C/P interviews were conducted by one author (PJL), an experienced clinical psychologist, who discussed all assessments with another author CC, a consultant clinical psychologist. Kappa for agreement on the presence/absence of diagnoses was 1.0. Table 2 contains a summary of participant diagnostic classifications.

### Analysis

Phenomenology seeks to understand individuals’ subjective experiences [45]. Given our aim to understand participants’ perceptions of barriers to access to targeted prevention, our philosophical base was experiential phenomenological [46]. We analyzed the data using Thematic Analysis [47], and report the study results in accordance with the consolidated criteria for reporting qualitative research (COREQ) [48]. We used the data-driven approach to coding and limited our focus in coding to the study’s research questions. We analyzed the data for adolescents as a group and separately for mothers as a group and used constant comparative techniques to code the data from the adolescent interviews, line-by-line and iteratively. Although transcribing each interview, these authors re-visited transcripts from any earlier interviews, reviewed their codes and updated these to incorporate their reflections and followed the same procedure for the mother interviews. We did not use specific computer software to facilitate coding or analysis. We gradually organized our codes into candidate themes and, where relevant, sub-themes. These themes were derived from the data. One author led the analysis, and met with other team members after each interview to discuss codes, tentative themes and possible interpretations of the data. Following Saldana [49], three authors (CC, KH and PJL) met to discuss and derive candidate themes and develop the finalized themes. This helped to promote the credibility and coherence of our interpretation of the data [50, 51]. We have used our final analytic structure to report cross-cutting themes within, and across, the participant groups.

## Results

Our analyses focused on participants’ views and experiences of potential barriers and facilitators to access to targeted anxiety prevention programmes from the perspectives of adolescents with anxiety disorders who, in infancy, were at risk of anxiety disorders and their mothers, who also had a history of anxiety disorders. Adolescents’ and mothers’ perspectives centred on three themes (see Table 3). We first report participants’ overall appraisals of whether they favoured targeted prevention (Desirability of targeted prevention); then examine their perceptions of barriers to targeted prevention (When and whether to act), and conclude with an examination of participants’ proposals for how prevention programmes could address barriers (Facilitators to access). We examined whether there were patterns in themes relating to the nature of participants’ risk factors (both BI and maternal anxiety disorder or only the latter), but found none.

**Table 3:** Themes

Super-ordinate themes	Sub-ordinate themes
1. Desirability of targeted prevention	1a. The right thing to do
	1b. Negative consequences of targeted prevention
2. When and whether to act	2a. When to intervene
	2b. Identifying anxiety as a problem
	2c. Responding to risk concerns
3. Facilitators to access	3a. Promote awareness
	3b. Practicalities of implementation

### Desirability of targeted prevention

1a. The right thing to do?

Participants expressed disparate views regarding whether targeted anxiety prevention was the ‘right’ thing to do. The principle of prevention appeared sufficient, for some mothers, to warrant taking up the opportunity of a targeted anxiety prevention programme: “I always think prevention is better than cure. Getting in early.” (M6); “I think that anxiety is so, so damaging. To me, it’s now, I see it as an illness as much as if someone broke their leg. And, of course, as a mum, if you could do something to prevent any of that happening, you would do it.” (M5); and “You’d do whatever you needed to not have the problems (develop).” (M2). While this favourable view of targeted prevention was common, one mother expressed concern about the principle of anxiety prevention: “Part of me says, actually, I think there’s a little voice saying ‘Are you trying to create perfect human beings?’” (M3).

The standing of anxiety prevention was an important feature when adolescents and mothers explained whether targeted anxiety prevention was the ‘right’ thing to do. Adolescents emphasized targeted prevention’s standing with their peers: “Maybe someone had this prevention programme and said ‘Oh this is—it did me no good’ then it would influence other people to think ‘Oh, maybe I shouldn’t do it’?” (A6). Mothers focused on the scientific standing of anxiety prevention: “Probably depends on where the research was at the time. Quite frankly, if it had been shown that early prevention is the better way to go, then yes.” (M3) and “Will this actually be positive or negative or have no effect at all? Why is an intervention needed—what are the pros and cons of it, versus not having it?” (M4).

1b. Negative consequences of prevention

Mothers were concerned by others’ stigmatising views of mental illness, and how targeted prevention programmes could lead to their children being stigmatized. While mothers stated that they did not hold stigmatizing views of mental illness: “to me, there’s no problem, no stigma. It’s like, if you’re prone to asthma or something like that, you deal with it.” (M7); they did express concerns about stigma from others. Notably, only mothers who had not sought treatment for their child’s anxiety disorder expressed concerns about negative consequences of prevention. Their concerns included others’ views of mental illness: “it’s a minefield, really,…if there is an issue, you point out the issue at school, who can then deal with it at an underhand level, so it means you’re not particularly pointing out the mental health.” (M2). These mothers expressed particular concern relating to stigma that their children could be treated inequitably, including by professionals: “I would still feel concerned if the teachers were aware. So ‘that child, that child and that child’—potentially are they then going to be dealt with or taught differently, which might be an issue.” (M5).

Adolescents’ expressed concerns about what their peers might think of them using a prevention programme: “I’d be okay with, like, my friends knowing but others round school I wouldn’t” (A4). Some mothers and adolescents expressed other reservations about targeted prevention related to concerns about possible unintended negative consequences. In particular, concerns focused on broader effects of prevention than altering a developmental trajectory away from clinically impairing anxiety: “I mean it could change me as a person completely and I don’t know if I’d want that.” (A2).

### When and whether to act

2a. When to intervene

Adolescents referred to two key issues to explain when they would have wanted a prevention programme: symptom severity and age. Those who referred to symptom severity consistently advocated that prevention would be relevant only once anxiety had begun to have a negative impact on them: “When you start feeling anxious, ‘cause before then you’re not really that bothered by it.” (A2). “I think I would rather have it while, or after, symptoms are showing ‘cause, beforehand, I might think ‘what’s the point of doing this?’. I probably wouldn’t pay attention to it”. (A7).

Age was a factor for some adolescents, although their views were mixed. Some thought that prevention at a young age might be excessive: “There’s no point giving to someone who’s younger with a bit of anxiety cos they might just…they might easily get over it”. (A1). Others, however, retrospectively valued prevention at an age before anxiety was problematic: “Well, I mean, if I, if when I was younger I could have it (prevention) and it would have prevented it (becoming a problem) and it would have been best, yeah; it would be alright, yeah.” (A6).

Mothers’ views of when they would have wanted a prevention programme were informed by their willingness to wait to see whether being ‘at risk’ progressed to signs of anxiety problems. While some mothers would have wanted prevention when risk factors were first identified: “if someone were to say to me from a very early age that there was the potential for problems, then I would very much have liked somebody to have stepped in at that point, and get things on a different path.” (M6); others would have taken a ‘watchful waiting’ approach: “(name removed) shows signs or symptoms and so on, I wouldn’t go straight away…but I’d wait to see if there was a pattern, or, you know, then see the GP.” (M4).

2b. Identifying anxiety as a problem

Mothers reported that it was difficult to know whether or not early signs of anxiety in their children were a cause for concern. Some mothers reported that they had not foreseen anxiety problems for their children: “I never anticipated anxiety to be present as a feature in her future life.” (M4), and went so far as to suggest that it might be their familiarity with their children that accounted for this: “As a mum, you think you know your child best, but you don’t always spot the problems because you’re living with them day to day.” (M5). Mothers reported that they felt unable to judge alone whether the signs were problematic. To help address difficulties with assessing signs of risk, one mother proposed the idea of a reference guide to signs of anxiety for them to monitor: “I would have been open to that—to a checklist of things to look out for at any point…like you do with any physical illness, really.” (M7). However, even where they might have had such a tool to help them assess their concerns, they had concerns about seeking professional input because they expected concerns about early signs of risk to be dismissed:

M2: “And you don’t wanna go down the GP and ask for anything,’cause you feel you’re going to get slated.

I: Oh!

M2: Yeah, well, er, yeah. ‘Cause normally when I go to the GP with anything they’ll say ‘Oh yeah, it’s normal.’

I: Ahhhh, okay.

M2: And you feel like a right idiot and a waste of time.”

2c. Responding to risk concerns

Mothers expressed their uncertainty about how they would respond to being alerted to signs as risks for anxiety problems. They spoke about feeling guarded: “Um, I’d be quite wary, I suppose. It’s almost like ‘Oh my goodness, what are they saying about my child?’” (M4); as well as the possibility of not knowing what to do: “There are a number of kids who are shy, whose parents are not, and have no clue how their poor introverted kid needs to deal with life.” (M6). Mothers emphasised the importance of their own and others’ experiences of successfully managing anxiety. This included the experience of their peers: “I do prefer if I know someone who’s been there and done it; I do like recommendations.” (M1); as well as professionals: “they (M6′s daughter’s nursery) knew exactly how her (M6′s daughter’s) mind works, and they were very good at dealing with her…if they can pick it up, then I’m sure they’re capable of saying ‘this is available.” (M6). One mother described her concern that if she had sought professional support with her son, then this might have exacerbated the impact of the anxiety: “You know, sometimes if you’re pointing it out to them (CYP), you’re making a problem worse, or you’re creating a problem.” (M2).

### Facilitators to access

3a. Promote awareness

Adolescents reported that absence of awareness of anxiety prevention programmes would be a crucial barrier to access. Adolescents were optimistic about the principle of anxiety prevention programmes, while emphasizing that their success is contingent on practical issues, including that people know that they exist: “the awareness of them kind of existing would be an issue…um, but if they were like prominent, if they were out there then I don’t think there would be as much as an issue?” (A5); and “it would be a really good idea to just like have this programme that’s known and talked about, people know where to go to, like, talk to someone to get it—they get the opportunity. I think it’s a really good idea.” (A7).

Mothers and adolescents suggested that schools could be involved in promoting awareness of anxiety prevention: “And I kind of think it’s become such a big issue now, with this age group, that I think perhaps if the school were to let them know that you might get periods in your life when you feel like this (clinically anxious)—and this is what you can do about it.” (M5). Adolescents specified how schools could play a role in promoting awareness of anxiety prevention, whether relatively passive: “I don’t want to say, like, advertisement, but putting up signs saying ‘If you’re worried (that anxiety is going to be a problem for you) then go and see, like, the school counsellor’ or go see, um, like quite an important person.”(A3); or via a relatively active approach: “I think school assemblies, like letters and that, and I think, maybe, somebody part of the programme coming into the school and talking about it?” (A7). Adolescents also stated that social media could helpfully promote awareness of anxiety prevention programmes: “also on social media as well I think that a lot of people…like everyone’s really influenced by social media at the moment obviously, um, so it would be good to sort of see it have more of a prominence there and to be more integrated onto um social media platforms.” (A5).

Adolescents spoke about the need to address stigma as a barrier, and open discussions could lead to acceptance: “generally people should talk more about it… just making sure, like, people realise it’s okay to get help and that they don’t have to sort of suffer on their own” (A5); “I think you need more acceptance to be honest” (A7).

3b. Practicalities of implementation

Adolescents and mothers focused on the format of targeted prevention programmes, including the mode and structure of delivery. We found differences within the adolescent group, and between the adolescents and mothers regarding the mode of delivery. Some adolescents saw contingent practical benefits for remote delivery: “It depends how far away. For example, if I had to drive 45 min for it, I’d be a bit like ‘I’d rather Skype’.” (A2); while others placed greater value on sessions in person:

A7: “Definitely a face to face chat, I think.”

I: “Why do you feel face to face?”.

A7: “Because an online resource…each person has a different thing, so an online resource isn’t going to be able to cover every possibility.”

This value of face-to-face contact was unanimously endorsed by mothers: “It’s probably a generational thing. You know, I’m more likely to be honest with somebody who I meet face to face. Um, (daughter’s name)—she’s constantly video-chatting to whoever.” (M3). Indeed, another mother emphasised the generational differences in acceptability of different treatment forms in response to the interviewer’s suggestion:

I: “…you could do it online…”.

M2: “Oh, that’s a nightmare.”

I: “A nightmare?”.

M2: “Especially for people like me—who are older”.

Mothers’ views encompassed where to deliver anxiety prevention, and identified potential benefits of schools. Mothers suggested school as suitable for a range of reasons, including necessity: “Some kind of education (for CYP at risk) at school. And it needs to start as soon as they go to juniors (age 7 years in England)” (M7), and opportunity: “I think it’s a question of going into the pre-schools and the nurseries and things like that.” (M6). One mother went so far as to propose that schools could be a first line location for targeted anxiety prevention “The child is in school for a good chunk of their life, so that is a good place to start, in fact. So, I think that if school had offered that facility, I’d have tapped into that.” (M4).

Adolescents and mothers focused on who should attend programmes. Some adolescents explained the importance of their parent(s) being involved in addition to themselves: “I think she (mother of A5) has had anxiety in the past but we’ve both had different types of it, kind of thing? And I think that by doing those sessions with her she’d be able to sort of recognise it and, um, understand it as well so we could both sort of work off it kind of thing.” (A5). For some adolescents, it was important that they and their parent(s) should have prevention sessions separate from each other so that they could speak freely: “If I was choosing to have it (prevention)—with my family—I would not want to have it in the same room as my Mum because I could never actually say what I’d want to say.” (A2); or to enhance the sessions’ usefulness: “I think it would be good to have it for you and your mum and dad but, like, separately…because that way the parents know about it so they can also know when to help in certain situations and that.” (A7). All mothers suggested that parents should be included in prevention. Some wanted to be involved with their children “I think parent and children together. But giving the parents the tools.” (M5); while some recognized the value of an option for parents to attend without children because: “Sometimes, I know the child doesn’t want it, do they? But I would have been open to give it a go.” (M7).

## Discussion

This is the first examination of possible barriers to targeted anxiety prevention with a sample of CYP who were at prospective risk of anxiety disorders in infancy and had gone on to develop anxiety disorders. This study illustrates the challenges posed for successful implementation of targeted anxiety prevention programmes, including the desirability of preventing anxiety disorders, when and whether to take action, and how to facilitate access to anxiety prevention programmes.

Mothers and adolescents held both positive and negative opinions about the desirability of targeted anxiety prevention, and expressed concerns about targeted prevention having unintended negative consequences, such as stigmatizing children and altering their personalities. This is consistent with the concern raised by Fox et al. [25] about whether effective prevention *should* be given to at risk CYP, given the economic and opportunity costs, and possibility of participation labelling a child and unnecessarily increasing parental concern. Certainly, no participants suggested that participation in targeted anxiety prevention programmes should be mandatory, allowing those with particular concerns to choose not to participate.

Regarding when to intervene, adolescent participants identified that *indicated* prevention might be preferable to *selective* prevention (i.e. prevention only once they experienced problematic anxiety symptoms, but not when they were pre-symptomatic). This is consistent with models of CYP help seeking for mental health problems. For example, Rickwood et al. [52] propose that young people’s help-seeking for mental health problems begins with their awareness of symptoms of mental health problems. Mothers, all of whom had experienced anxiety disorders prenatally, reported difficulties in recognizing early signs in their children of anxiety as risk factors for development of anxiety disorders. This is consistent with earlier literature regarding the impact of parent anxiety on their children’s use of clinical services [30, 53, 54]. For example, Jongerden et al. found that higher maternal anxiety was significantly associated with lower likelihood of their child being referred for the treatment of their anxiety disorder. Mothers in the present study also pointed to the importance of their expectations of being dismissed by health professionals if they were to raise concerns about their children’s anxiety. This is consistent with the literature regarding anxiety as well as other mental health conditions, where this parent concern deterred help seeking [55]. Mothers also expressed concerns about making their child’s anxiety worse by pointing it out, which was a concern expressed by over a quarter of parents in an implementation study of anxiety treatment in Australia [56].

Regarding the practicalities of delivering anxiety prevention programmes, particularly to whom sessions are offered, all mothers wanted to be included in the prevention programmes, and adolescents identified benefits to their parents having sessions separate from themselves. This involvement of parents is consistent with approaches taken in the previous anxiety prevention programmes for young children, in which sessions are typically offered to parents, but not their young children [e.g. 24–26].

### Implications

We identified possible ways to facilitate access to targeted anxiety prevention. Adolescents and their mothers highlighted the important roles schools could play in supporting targeted anxiety prevention. Previous studies have identified schools as being crucial to the success of prevention of depression [57] and in supporting families of anxious children who have not gained access to professional services [31]. In the present study, adolescents particularly highlighted a potential role of schools in promoting awareness of anxiety prevention programmes, and mothers joined them in suggesting that schools could be instrumental in delivery of anxiety prevention. Indeed, RCTs conducted in schools in Australia [58], Europe [59] and North America [60], have shown that, as compared to controls, targeted prevention can effectively reduce anxiety symptoms. However, these were efficacy studies, not designed to examine routine involvement of schools. Future studies should examine the feasibility of schools routinely (i) screening children for risk of anxiety disorders (as has been done in the USA [61]) and (ii) delivering targeted anxiety prevention programmes. In addition, adolescents suggested that social media could play a helpful role in promoting awareness of anxiety prevention. We know of no studies examining the use of social media to promote access to intervention or prevention of CYP anxiety disorders, but the value of social media has been identified in promoting access to clinical services for CYP more broadly [62].

Mothers and adolescents reported that the severity of anxiety signs and symptoms were important determinants of when to intervene, while only adolescents referred to age as a key factor. The format of prevention was also important to adolescents and their mothers. Although adolescents recognized possible benefits of remote and online anxiety prevention programmes, mothers reported being more ‘traditional’ in wanting to meet professionals in person. A possible explanation for this is that adolescents are digital natives, and are more comfortable with digital technologies than are their parents [63]. Future studies could test this by examining the opinions of parents (of young children at risk of anxiety; that is, those who would be the targets of prevention and perhaps a decade younger than the parents in the current study) regarding the use and appeal of online anxiety prevention resources.

Our findings are distinct from the two extant studies that reported barriers to access to targeted anxiety prevention [26, 27]. In those studies, parents had signed up to participate in randomized controlled trials of prevention programmes, and so had managed to overcome potential barriers to initial access. They reported barriers which were predominantly practical: lack of childcare, inability to take time off work and travel difficulties. In contrast, mothers in our study reported less tangible issues, such as stigma [64] and wariness about their children being identified as at risk, as well as the principle of prevention and its scientific standing. Future studies might fruitfully examine whether these differences arise from (i) the characteristics of different groups (for example, a group comprising families who had sought and received prevention, in comparison to a group who had neither sought, nor received, prevention) or (ii) other factors (based on our findings, these might be beliefs about stigma or the scientific standing of prevention), or a combination of these. Research could then use this information to examine how to make effective prevention available and appealing to families at risk; for example, by tackling a tendency not to seek prevention and by addressing stigma associated with mental illness.

### Strengths and limitations

We used a prospective longitudinal study to obtain our participant pool, yielding a unique sample for this study of families where infants were prospectively identified as being at risk of anxiety disorders, and had then gone on to develop these. This gives us high sample specificity [42]. We used diagnostic assessments with all participants, confirming that anxiety caused significant impairment in their lives, as well as observational assessment of behavioural inhibition in infancy. Despite these strengths, we must outline our study’s limitations. Many families from earlier waves of the longitudinal did not respond to invitations to participate in the present wave of data collection, and so were not part of our pool of potential participants. It is likely therefore that our sample lacked diversity in important characteristics, also, the sample was exclusively White British, and recruited from a single county in England. Future examinations must seek to understand the experiences in more ethnically and socio-economically diverse families. Information Power [45] has been criticised for possible features of pragmatism [65] and we cannot demonstrate that our sample size was unaffected by these features. Methodologically, one of the authors had conducted diagnostic assessments with participants as part of the longitudinal study. Thus, he had an established relationship with participants before interviewing them for the present study, and this will have affected the interview data. Further, two authors are clinical psychologists, so might have a disposition to hold a positive view of the principle of targeted anxiety disorder prevention.

This study emphasizes the difficulties that might impede the implementation of targeted anxiety prevention programmes, including difficulties in recognizing risk factors and concerns regarding being dismissed by health professionals. It also identifies possible facilitators to access to prevention, including promoting awareness of such programmes both in schools and on social media. Closer examination of barriers and facilitators to prevention is needed with children who are at risk, but have not yet developed anxiety disorders.

## Supplementary Information

Below is the link to the electronic supplementary material.

Supplementary file1 (DOCX 35 KB)
