# Supplementary material for: Barriers and facilitators to targeted anxiety prevention programmes in families at risk: a qualitative interview study
Source: Eur Child Adolesc Psychiatry. 2020 Dec 21;31(4):565–75. doi: 10.1007/s00787-020-01703-4 (PMC9034995; doi:10.1007/s00787-020-01703-4)
Supplement: Supplementary file 1 — Supplementary file1 (DOCX 35 KB) [file 787_2020_1703_MOESM1_ESM.docx]

**Participant flow diagram**

Pregnant mothers assessed for anxiety disorders from 20 weeks

(n=427)

1999-2004

Infants assessed for

Behavioural Inhibition at 14 months

(n=217)

2001-2005

Adolescents assessed for anxiety disorders aged 14-17 years

(n=34)

2016-2018

Qualitative interviews with adolescents (n=7) and mothers (n=7)

2017-2018

**A table showing which participants contributed to which sub-themes.**

| Sub-ordinate themes | Contributing participants |
| --- | --- |
| 1a. The right thing to do | A6.  M2, M3, M4, M5, M6. |
| 1b. Negative consequences of targeted prevention | A2, A4.  M2, M5, M7. |
| 2a. When to intervene | A1, A2, A6, A7.  M4, M6. |
| 2b. Identifying anxiety as a problem | -  M2, M4, M5, M7. |
| 2c. Responding to risk concerns | -  M1, M2, M4, M6. |
| 3a. Promote awareness | A3, A5, A7.  M5. |
| 3b. Practicalities of implementation | A2, A5, A7.  M2, M3, M4, M5, M6, M7. |

**Patient and Public Involvement (PPI)**

We first discussed our idea for this project at a PPI meeting at the University of Reading, UK in mid-2016. (This was a regular meeting of adolescents who had used local Child and Adolescent Mental Health Services (CAMHS) and their parents to discuss research projects from early ideas, to those nearing dissemination.) At that meeting, PJL was able to discuss the project with adolescents who reported having experienced anxiety disorders, as well as their parents. These PPI group members guided PJL to conduct the study using individual interviews (rather than group or dyad interviews) and to examine not only what study participants would want in targeted anxiety prevention programmes, but also what they believed were the barriers and possible facilitators to access to such programmes. They also offered guidance on the first draft of the Topic Guide.

The second time we conducted PPI work was in late 2016. PJL and CW met with a young adult who had experienced mental health difficulties and was experienced in helping to develop and guide research studies with adolescents with anxiety and depression. On this occasion, the focus of the PPI work was to finalize the Topic Guide.

**Topic Guides**

Adolescents

When should something be done (what would help / hinder)?

With whom (self, parents, family, professionals, friends)?

What want & How delivered (what would help / hinder)?

What to expect?

Awareness (what would help / hinder)?

Importance of others (as barriers or facilitators)?

Mothers

When should something be done (what would help / hinder)?

With whom (self, parents, family, professionals, friends)?

What want & How delivered (what would help / hinder)?

What to expect?

Awareness (what would help / hinder)?

Importance of others (as barriers or facilitators)?

Responses to being at risk (e.g., what expect from GP?, School? Noticing signs, access to services)?

**Use of Field Notes**

PJL and CW each kept field notes from the interviews they conducted. They used these notes to provide context for their discussions of transcripts and coding. For example, PJL used their field notes in supervision with CC when discussing mothers’ views of whether or not prevention is desirable. Both CC and PJL are clinical psychologists and, as such, likely have biases regarding prevention of mental health difficulties being preferable to their realization. With this possibility of bias in mind, PJL used their field notes to be able to share information with CC beyond the transcripts, such as PJL’s thoughts and feelings during the interviews.

PJL kept field notes during data analysis, including written notes, and photographs. They used the written notes as a record of i) what each code encompassed and, when there was possible overlap, ii) why an utterance was coded as one thing rather than another, and iii) why we (the authors) revised our themes. They used the photographs to record proposals for how codes might comprise themes (that is, to show the development from our initial ideas for themes - with supporting codes - to those we ultimately chose – with *their* codes).
